# Celluloid

**Published:** 1877-04

**Authors:** 


### ARTICLE III.

### *Celluloid.*

To the Dental Profession.—In reply to numerous inquiries as to whether the recent decision of the Supreme Court at Washington, in the case of "Smith *vs.* Dental Vulcanite Company," affects the use of *Celluloid*, we would state that the said suit was brought against Dr. Smith for his use of Rubber in making dental sets, and that the decision therein does not in any way affect the question of the right to use *Celluloid.*

The decision as printed is too long to be given in this circular, embracing as it does over sixteen pages of a large pamphlet, but we quote some extracts in order to show conclusively that not only was *Celluloid* not included in the case, but that the language of the decision is entirely in favor of limiting the "Cummings Patent" *exclusively to Rubber.*

Mr. Justice Strong, in delivering the opinion of the Court, after giving a brief history of the applications for and issuing of the "Cummings Patent," goes on to say: "If then the claim be read as it should be in connection with the preceding part of the specification, and constructed in the light of the explanation which that gives, the invention claimed and patented is a set of artificial teeth as a new article of manufacture, consisting of a *plate of hard rubber* with teeth, or teeth and gums secured thereto in the manner described in the specification, by 'imbedding the teeth and pins in a *vulcanizable compound, so that it shall surround them while in a soft state before it is vulcanized,* and so that when it has been vulcanized the teeth are firmly and inseparably secured in the vulcanite." * * * "It was well described by the Circuit Judge as the 'making of a *vulcanite dental plate* out of a *vulcanizable compound into which the teeth were imbedded in its plastic condition,* and the rubber compound, with the teeth thus imbedded in it, *afterwards vulcanized by heat,*' * * * so that the plate should possess the quality of hard rubber."

In conclusion, the Court found that the patent was a valid one, and therefore the decree of the Circuit Court was affirmed.

This decision settles finally the question of the validity of the "Cummings Patent," so far as the use of *Rubber* is concerned, and the only way in which the profession can escape the yoke of the "Goodyear Dental vulcanite Co." is in continuing their use of "CELLULOID." It has been reported to us that the Vulcanite Company, through Mr. J. Bacon, their treasurer, has claimed, from time to time, that decisions had been rendered in their favor by various Courts in regard to "CELLULOID." In point of fact only one case in which we have appeared has ever reached a decision (G. D. V. Co. *vs* E. M. Flagg). The motion was for a preliminary injunction, *and was decided in our favor.* The G. D. V. Co then declined to go on with the case, but proceeded to bring suits against dentists in different parts of the country for their use of "CELLULOID," thereby hoping to frighten dentists into paying them license fees for such use. Wherever we were notified of such suits we have assumed the defence, and are now so defending a large number of dentists. Of these suits none have ever reached a hearing, with the exception of a test case which was tried in the latter part of October, 1876, before Mr. Justice Shepley, at Boston. At the close of the argument permission was given the counsel for the respective parties to file briefs. The briefs on our part were filed on the 20th day of November last, but the reply of the complainant's counsel has only been filed within the past week, although it was understood between the counsel that they should be filed by the 1st day of December last.

In this way a decision has been delayed, the time having been availed of by Mr. Bacon, treasurer, to distribute broadcast circulars conveying a false impression as to the effect of the decision in the " Smith Case." In one of such circulars now before us, dated January 10th, 1877, referring to this decision, he says: "Thus has the controversy between this Company and your profession been finally settled by the Court of last resort *in favor of this Company*," whereas the only point really decided was that the "Cummings Patent" was valid so far as a dental plate of VULCANITE, made according to the process therein described, was concerned.

We ask the dentists to put faith in our ability and willingness to defend them in the use of our base, and to refuse to pay any license fee for such use. The very fact that the "Goodyear Dental Vulcanite Company" are now offering extra inducements to dentists to pay up their license fees *immediately* shows weakness and a desire to secure the money, *before the Court, in the test case which has been tried, renders its decision*, which they have good reason to fear will be against them.

They are also in some cases, agreeing to refund the money so paid for license fees, provided the decision in said case be against them; but we would like to ask the dentists whether any thing in their past dealings with Mr. Bacon, treasurer, would lead them to believe that they are likely to recover the money so paid, except as the result of a suit, which would cost more than the amount at issue.

Yours respectfully,

THE CELLULOID MANUFACTURING CO.

Newark, N. J , Feb. 28, 1877.

